# Diet Implications and Oral Health Status of Women in Central Italy

**DOI:** 10.1055/s-0041-1735906

**Published:** 2021-12-10

**Authors:** Giulia Zumbo, Micaela Costacurta, Francesca Zara, Nicola Pranno, Margherita Ceravolo, Francesco Covello, Matteo Saccucci, Iole Vozza

**Affiliations:** 1 Department of Oral and Maxillo-Facial Sciences, Sapienza University of Rome, Rome, Italy; 2 Department of Surgical Sciences, University of Rome Tor Vergata, Rome, Italy

**Keywords:** oral hygiene, oral health, diet, oral habits, nutrition, omnivorous, vegetarian, vegan, women, Italy

## Abstract

**Objective** The relationships between oral health habits, dietary practices, and oral health status, as well as general health status, in the population of Italian women, are complex, with many mutual and interrelating factors. The purpose of this study is to investigate oral habits, oral status, and dietary habits of a sample of women in Italy, highlighting the links between nutrition and oral health and discussing how oral health care professionals can integrate nutrition counseling that aimed at improving the oral health of their patients into their practices.

**Materials and Methods** In the period between February 2020 and July 2020, an anonymous questionnaire made up of 20 questions was administered to a randomized sample of 120 Italian women.

**Results** Most of women declare good manual skills in oral hygiene (50%) but just less than half of them brushes their teeth more than three times a day. Statistically significant correlations were found between frequency of dental checkups and dental mobility ( *p* = 0.036), and halitosis ( *p* = 0.006), as well as between frequency of flossing and gum bleeding. Correlation between the type of diet and oral health status showed more halitosis (∼21%), sensitivity (∼26%), and xerostomia (∼53.3%) for vegetarian and vegan women.

**Conclusion** More awareness need to be raised concerning oral hygiene habits, and regarding the importance of regular dental checkups. Brushing at least twice or three times a day needs to be encouraged and the valid support of dental aids has to be in the everyday domestic oral hygiene protocol, as scientific evidence demonstrates. Future clinical studies need to be performed on a more consistent number of vegetarian and vegan patients, to obtain more statistically significant results and support future research that will compare omnivorous, vegetarian, and vegan diets and their influence on oral health status.

## Introduction

The relationships between oral health habits, dietary practices, and oral health status, in the population of Italian women, are complex. Indeed, there are many mutual and interrelating factors which play an important role. Just as inadequate nutrition can affect oral health, poor oral health status affects food choices and, thus, nutritional status. 1 It is clearly essential that the primary care practitioner and/or screening health professionals always include an evaluation of oral status in the assessment of general health of a patient. Moreover, dentists and dental hygienists are in the unique position to provide both oral hygiene instruction and basic nutrition information related to oral health. Unfortunately, many patients may not be aware of the effects of diet and nutritional status on the development and maintenance of a healthy mouth and caries-free teeth. Therefore, it is vitally important for these professional figures to perform basic nutrition screening to assess clients' dietary habits for potential risk factors for dental caries and periodontal disease. Collaboration between dietetics professionals and oral health care professionals is essential in identifying, educating, and treating oral health problems related to nutrition. Such partnerships will encourage improved levels of oral health care. 2

However, one health challenge is maintaining a natural and healthy dentition throughout the life span 3 4 5 6 7 because this has a considerable impact on the chewing function. In addition, chewing is also a critical first step in oral processing of food materials for nutrient procurement. 8

In conclusion, an individual's oral health status has a profound impact on the intake and utilization of nutrients. Moreover, interchangeable nutrients tend to determine the state of oral health by preventing tooth loss and oral diseases. In view of this, the purpose of this study is to highlight the link between nutrition and oral health in a sample of women in Italy and discuss in which way oral health professionals can integrate nutrition counseling.

## Materials and Methods

In the period between February 2020 and July 2020, an anonymous questionnaire, made up of 20 questions, was administered to a sample of Italian women, aged between 15 and 60 years. The questionnaire was prepared and administered through the “Google Forms” platform and posted on social network groups (Facebook) in Italy.

The questionnaire was prepared together with a statistician that established the sample size sufficient for study validity. Among the 400 surveys received, 44 were incomplete; among the remaining 356, an established sample of 120 subjects was randomized through an algorithm generated though the SAS Software (Analytics Software & Solutions) (SAS Inc., United States, 2020).

The study protocol complied with the Guidelines for Good Clinical Practice, according to the Declaration of Helsinki (1975). The study was approved by the Institutional Review Board of territorial NHS facilities (no. 3003/19).

The 20 questions, in the questionnaire, were divided into two parts. In the first part, there were questions that aimed at collecting information regarding their age, their type of diet, and the period when they embraced their current dietary style. In the second part, questions regarding their oral hygiene, dental and periodontal health (presence of bleeding, mobility, sensibility), and frequency of dental checkups were asked.

### Statistical Analysis

The data gathered were recorded with a specially designed computer program and collected in a Microsoft Excel database. Data were evaluated using standard statistical analysis software (version 20.0, Statistical Package for the Social Sciences, IBM Corporation, Armonk, New York, United States). Descriptive statistics including percentage and frequency values were calculated for each variable. The relationship between the following variables was explored:

- Type of diet and bad breath
- Type of diet and sensitivity
- Type of diet and bleeding
- Type of diet and dry mouth
- Number of checkup visits and dental mobility
- Number of checkup visits and gum bleeding
- Number of checkup visits and bad breath
- Use of dental floss and gum bleeding
- Use of dental floss and bad breath
- Use of interdental cleaning brush and dental mobility
- Type of toothbrush and gum bleeding.

For all categorical variables, group comparisons were assessed by chi-square test of homogeneity, if expected cell frequencies were lower than five, Fisher's exact test was used. Phi (ϕ) measures were used to evaluate the effect size of a nominal-by-nominal relationship. The effect size of each association was evaluated according to the following conversion table:

Score from 0.1 to 0.20: weak association;

Score > 0.20 to 0.30: moderate;

Score > 0.30: strong.

A *p* -value of ≤0.05 was considered as statistically significant.

## Results

In the period between February 2020 and July 2020, 120 questionnaires were collected. All of them were properly filled and none was excluded.

Results are presented in accordance with a first descriptive evaluation of parameters followed by a correlation analysis between some of them. Among the data collected from the questionnaires and then submitted to statistical analysis, there are many statistically significant associations. However, some results with a nonstatistical *p-* value ( *p* -value <0.05 as significant) were chosen to be reported, considered important for the purpose of this study.

Descriptive analysis shows a female population, and the majority (73%) is 36 to 55 years old and follows an omnivorous diet ( Table 1 ). Most of the women declare good manual skills in oral hygiene (50%) but just less than half of them brushes their teeth more than three times a day ( Table 2 ). Regarding oral hygiene habits, a great part uses a whitening toothpaste (18%). The prevalence in use of dental floss, interdental cleaning brush, brush tongue cleaning, and mouthwash is described in Table 3 .

**Table 1:** Different kind of diet followed

	Frequency ( *N* )	Percentage (%)
Omnivorous diet	85	70.8
Vegan diet	7	5.8
Vegetarian diet	28	23.3
Total	120	100.0

**Table 2:** Frequency of brushing

	Frequency ( *N* )	Percentage (%)
1	11	9.2
2	60	50.0
>3	49	40.8
Total	120	100.0

**Table 3:** The prevalence in the use of dental floss, interdental cleaning brush, brush tongue cleaning, and mouthwash

	Frequency ( *N* )	Percentage (%)	Cumulative percentage (%)	Frequency ( *N* )	Percentage (%)	Cumulative percentage (%)	Frequency ( *N* )	Percentage (%)	Cumulative percentage (%)	Frequency ( *N* )	Percentage (%)	Cumulative percentage (%)
	Frequency in using dental floss			Frequency in using interdental cleaning brush			Frequency in using brush tongue cleaner			Frequency in using mouthwash		
Everyday	17	14.2	14.2	4	3.3	3.3	6	5.0	5.0	6	5.0	5.0
Often	32	26.7	40.8	12	10.0	13.3	10	8.3	13.3	22	18.3	23.3
Sometimes	32	26.7	67.5	27	22.5	35.8	18	15.0	28.3	45	37.5	60.8
Almost never/never	39	32.5	100.0	77	64.2	100.0	86	71.7	100.0	47	39.2	100.0
Total	120	100.0		120	100.0		120	100.0		120	100.0	

Among all the women who filled the questionnaire, only 4.2% declare a constant presence of bleeding after brushing teeth, while 9.2% say they have dental sensitivity. On the other hand, the same percentage of women (0.8%) declares to have bad breath and dental mobility. All the results reflect a population of women who, for ∼55%, did not use to undergo very often to dental checkups. Indeed only 29% of all women went to the dentist every 6 months.

Regarding statistical correlation between different parameters, it is appropriate to start mentioning data correlated with diet, in accordance with the focus of the article. Data reveal that among all women who completed the questionnaire, even though the correlation is not statistically significant ( *p* -value <0.05 as significant), the relationship shown between the type of diet and several oral symptoms is interesting. The only case in which results reveal the constant presence of bad breath is when women follow a vegetarian diet; on the other hand, the omnivorous diet shows the majority percentage of women with no halitosis problems (75.4% omnivorous women reporting not to have it vs. 18 and 6.6% of vegetarian and vegan, respectively). However, according to dental sensitivity, the major percentage (54.5%) of women who always feel it is present in women who follow a vegetarian diet, while omnivorous diet reveals the highest score of no sensitivity at all (74.3% of omnivorous vs. 20 and 5.7% of vegetarian and vegan not having it).

**Fig. 1:**
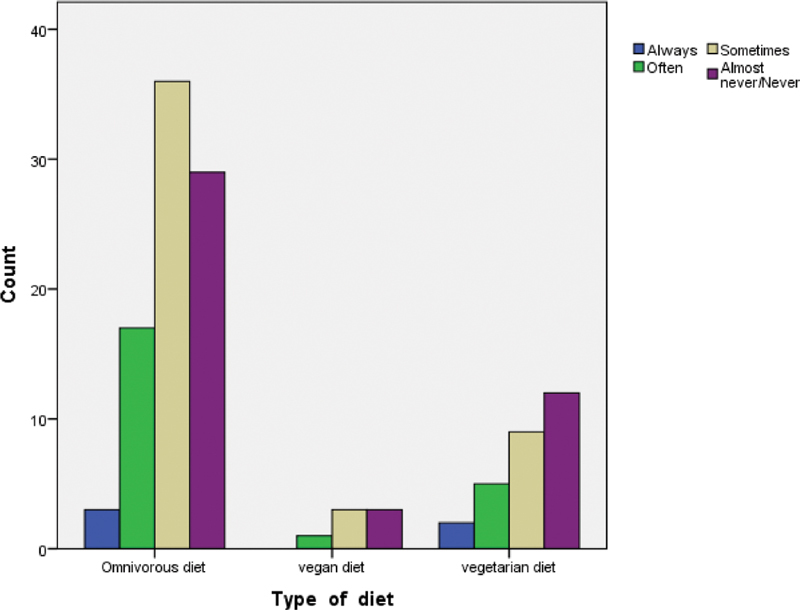
Correlation between kind of diet and gum bleeding.

Regarding gum bleeding, among the omnivorous women, low percentages were reported to be suffering from it always or often (3.5% always and 20% often). Similar was for vegetarian women (7.1% always and 17.9% often) and for vegan women (0.0% always and 14.3% often) ( Fig. 1 ).

**Fig. 2:**
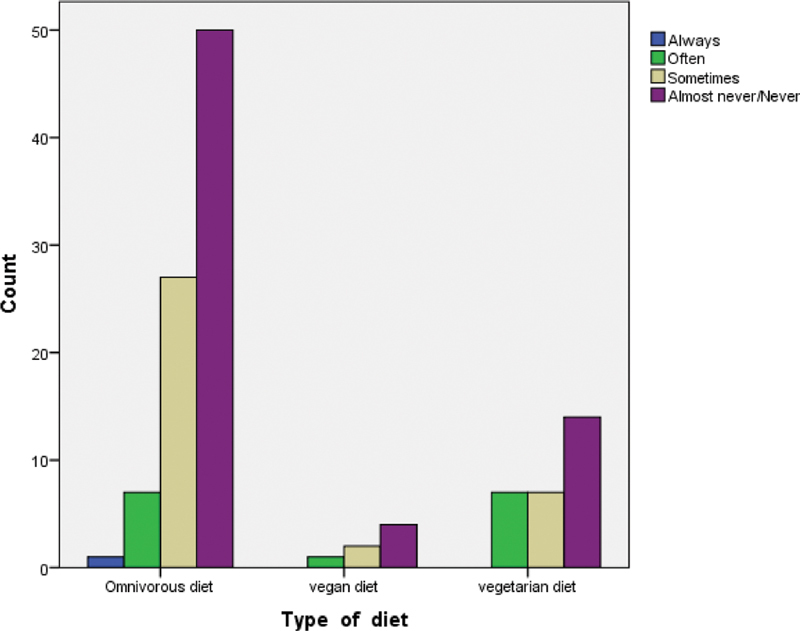
Correlation between kind of diet and mouth dry.

In addition, among the interviewed women, very few of them (12.5%) reported to be often feeling dryness in the mouth (xerostomia), among these, 46.7% were omnivorous, another 46.7% were vegetarian, and the remaining 6.6% were vegan ( Fig. 2 ).

The first moderate association (ϕ = 0.227) with significant *p* -value ( *p* = 0.036) was obtained between dental mobility and gum bleeding with checkup visits. In fact, 71% of women, who had undergone dental examination, did not reveal dental mobility, while only 0.8% showed an evident mobility of teeth. On the other hand, 38% of women, who followed frequent checkup visit, did not show gum bleeding compared with a small percentage (4%) with gingival inflammation.

**Fig. 3:**
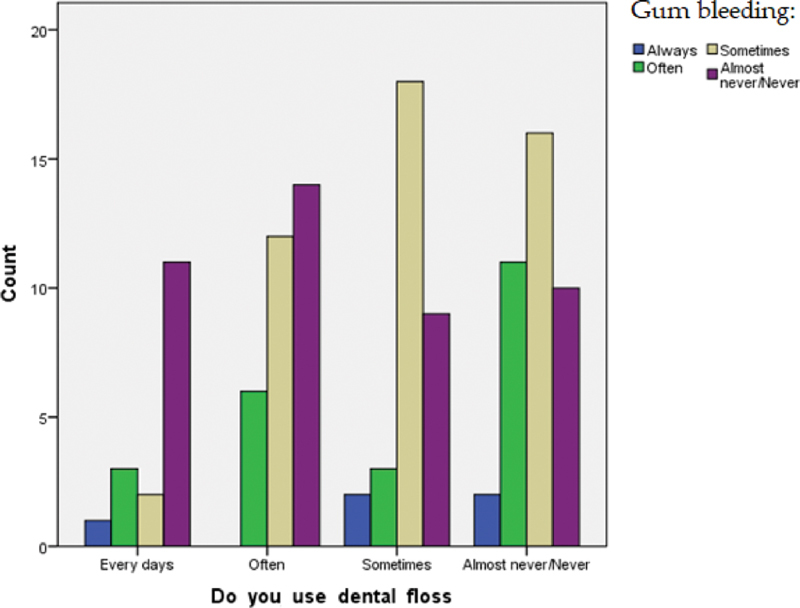
Correlation between flossing and gum bleeding.

**Fig. 4:**
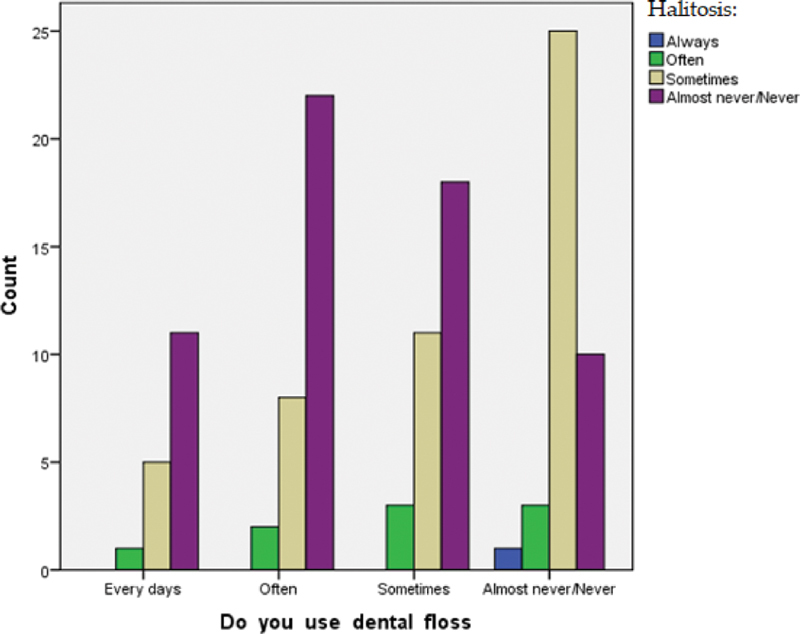
Correlation between flossing and halitosis.

Another moderate statistical association (ϕ = 0.278; *p* = 0.034) exists between the use of dental floss with gum bleeding and bad breath after brushing the teeth. In fact, among women who use dental floss, 40% have just sometimes showed gum bleeding, while 37% never suffered from this problem ( Fig. 3 ). Otherwise, considering the correlation with bad breath, data show that 41% of women felt bad breath despite the use of dental floss versus 51% who almost have never had this discomfort ( Fig. 4 ).

In addition to dental floss, there is a strong statistical correlation (ϕ = 0.441) also between interdentally cleaning brush and dental mobility ( *p* = 0.015). Indeed, 50%, among who often use interdentally cleaning brush, had no dental mobility; the remaining 50% also had low teeth mobility. Otherwise, among the ones who have never used this device, 42% had no dental mobility and only 0.8% reported a considerable mobility of teeth.

**Fig. 5:**
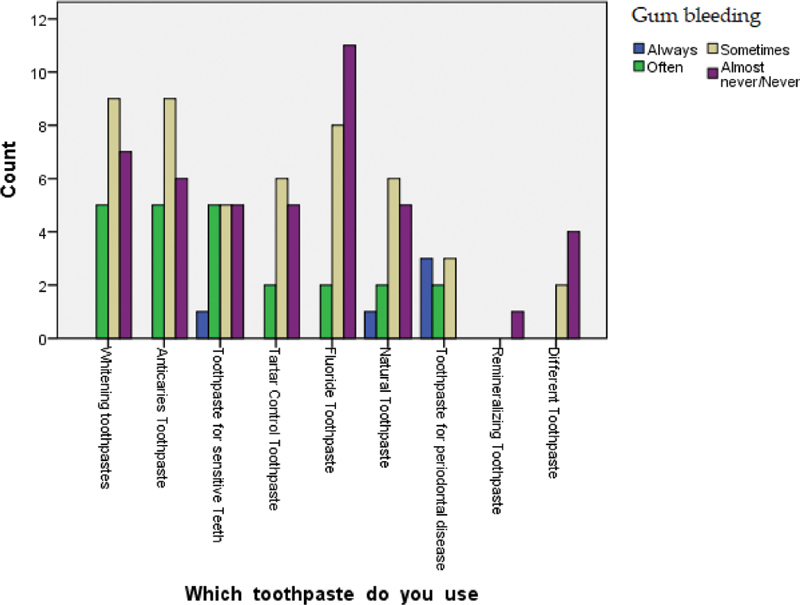
Correlation between type of toothpaste and gum bleeding.

Looking at the association between the kind of toothpaste and the presence or absence of gum bleeding, data reveal different percentages. The toothpaste for sensitive teeth, together with natural toothpaste, showed the highest percentage in association of gum bleeding (20%) followed by toothpaste for periodontal disease (2.5%). Instead, tartar control toothpaste obtained the highest score (39%) in no bleeding among all toothpastes analyzed. Fig. 5 shows, in more detail, the results obtained.

Looking at other statistically significant data, results reveal that among who follow checkup visits every 6 months, 19 and 24% have almost never had bad breath (ϕ = 0.512; *p* = 0.006) and dental mobility (ϕ = 0.318; *p* = 0.021), respectively. Among the ones undergoing dental checkups twice a year, 82.9% declared not to be having dental mobility, as well as 77.8% who undergo dental checkups once a year; the percentage dramatically drops in women who never go to the dentist, rising on the other side the percentage of reported dental mobility (66.7%).

Age, different kinds of toothbrush, and mouthwash reveal no statistical correlation but, at the same time, it is interesting analysis of some specific characteristics. For example, the toothbrushes with medium bristle are associated with the highest percentage (53%) of no dental mobility. In addition, despite the values are not statistically significant, there is also increasing bleeding and bad breath in the ones who brush their teeth only once, when compared with the ones who brush their teeth at least three times a day.

## Discussion

This study aimed to find a link between nutrition and oral health in a sample of women in Italy and discuss in which way oral health professionals can integrate nutrition counseling that affects food choices and, thus, nutritional status.

Unfortunately, this topic is poorly discussed in literature, so it was hard to make a great comparison between results obtained and what literature explains.

However, starting from how aliments and diet influence oral health, different studies face the association between the amount and frequency of free sugar intake and dental caries. 9 10 11 Although other fermentable carbohydrates may not be totally blameless, epidemiological studies show that the consumption of starchy staple foods and fresh fruit is associated with low levels of dental caries. In addition, the frequency of consumption of foods containing free sugar should be limited to a maximum of four times per day. 9

Otherwise, regarding different types of diets, it seems that the number of people embracing a vegetarian lifestyle is constantly growing. Indeed, studies involving countries all over the world produced variable results of vegetarianism prevalence among the general population: 0.77% in China, 0.79% in Italy, 1.5% in Spain, 3.3% in Germany, 3.8% in Norway, 4.1% in Finland, from 3 to 5% in Latvia, 11.2% in Australia, 33% in Southern Asia, and from 4.8 to 5.6% in Sweden. 10 Vegetarianism implies a dietary regime, or conception, lacking meat, poultry, or fish. There are many reasons, related to health, ethical, social, or environmental motivations, that lead to take up a vegetarian diet. 11 Veganism, as well, is generally adopted to carry out ethical principles related to animals' rights and well-being, but also to comply with spiritual, moral, and religious values, for socioeconomic considerations and for environmental reasons, supporting concepts such as energy saving and the use of natural resources in food production. 12 13 14 15 16 17

Vegetarian diets are characterized by different nutritional models according to the exclusion of one or more type of food. The most common ones are the lacto-ovo vegetarian diet (it excludes meat and fish, but includes dairy, eggs, and honey, together with a great variety of vegetal food), the lactovegetarian diet (– it excludes meat, fish, and eggs, but includes dairy, honey, and great variety of vegetal food), the ovovegetarian diet (it excludes meat, fish, and dairy, but includes eggs and honey, together with great variety of vegetal food), and the vegan diet (it excludes meat, fish, dairy, eggs, and honey, but includes a great variety of vegetal food). 14

Particularly, scientific evidence suggests vegetarian diet carries out a positive impact on body mass index, cholesterol levels, glucose levels, and risk of cardiovascular diseases and cancer. 18 However, to this day, it is not clear if a vegetarian diet may have effect on oral cavity health. Several associations have been made between diet and oral health, such as the connection between the consumption of sugar and the onset of caries or periodontal problems. 19 20 21 Nevertheless, studies that focus on the association between the type of diet and diseases in the oral cavity are not many. 22 Since the 1970s, some studies have examined possible connections between vegetarianism and dental health, 19 analyzing the correlation of acid food consumption and dental erosion. Scientific evidence from the most recent studies shows variable results; while some found positive associations, others found negative ones. 23 24 Our study showed how in this sample of women, the most common diet is the omnivorous one (70.8%). Indeed, results showed that, when compared with women following vegetarian diet, there was less incidence of bad breath (75.4% omnivorous women reporting not to have it vs. 18 and 6.6% of vegetarian and vegan, respectively). Even though the correlation is not statistically significant, this is probably due to the difference in number of the groups: omnivorous, vegetarian, and vegan; further investigations with more homogenous samples will be interesting to research to affirm a certain factor in vegetarian and vegan diet that would support the higher presence of halitosis. Moreover, for future research, differential diagnosis among physiological halitosis, pathological halitosis, pseudo halitosis, and halitophobia could be performed through different methods (organoleptic evaluation, gas chromatography, and salivary β-galactosidases activity). 25 26 This can be diriment in understanding if a connection between halitosis and vegetarian diet exists. The same kind of reasoning goes for the sensitivity aspect, for which higher percentages in omnivorous women declared not to have it (74.3% of omnivorous vs. 20 and 5.7% of vegetarian and vegan, respectively). For this reason, it could be interesting for future research purposes to investigate the etiopathology of this hypersensitivity.

Concerning gum bleeding, there are no studies in the literature which aim to examine it. From our results, no significant difference was recorded for the different types of diet, with relatively similar and low percentages reported in the three groups, suggesting that there may not be a correlation between diet and gum bleeding.

The last aspect analyzed regarding xerostomia and diet showed equal percentages for omnivorous and vegetarian women, and a lower one for vegan, but no significant correlation between the two variables. This might suggest that the difference in the diets does not influence the sensation of dryness in the mouth and that the lower percentage of reported xerostomia might be related to the poorer numerosity of the women following that type of diet.

For what that concerns the aspect of vegetarian subjects' oral health, some mechanisms that might explain the potential associations between vegetarianism and dental health have been proposed. Even if at this day, it is still controversial, some results in scientific literature show potential evidence related to the risk of dental erosion in people following a vegetarian diet versus the ones not following it. People exclusively assuming vegetable food tend to eat more fruit and vegetables compared with people following a nonvegetarian diet, therefore introducing more acid foods that cause a reduction of the salivary pH. 19 To this day, there are still few scientific data available regarding the correlation between vegetarian diet and oral health. Some epidemiological studies suggest a multifactorial etiology for white lesions of dental enamel (demineralization), erosion, and abrasion, for which food habits can play a significant etiological role.

Another interesting data emerging from literature is the absence of caries in individuals following a vegetable-based diet, most likely connected to the consumption of fresh fruit during lunch that demonstrated to be significant in self-cleansing. In fact, among the individuals exclusively eating vegetable-based food, a lower incidence of gingival tissues inflammation was observed. 23

Time also resulted to be another important factor to be considered. In fact, potential harmful factors for dental health may show up more probably in subjects who follows this kind of diet since birth rather than in the ones following it for few months or years. 19

This study, as well as other present in literature, 19 27 28 confirms the good oral hygiene habits of vegetarian and vegan subjects.

Moving onto the topic of oral hygiene habits, it is widely known that good oral hygiene practices are broadly considered important in maintaining good oral health, and flossing has long been considered an indispensable part of an effective oral hygiene routine. 29

Our study showed statistically significant results ( *p* = 0.034) that strengthen the association between flossing and healthier gums, as 37% never suffered from this problem and 40% have just sometimes showed gum bleeding using floss or interdentally brushes in addition to tooth brushing may reduce gingivitis or plaque, or both, more than tooth brushing alone. 30 31 32 Therefore, as in some cases, these conditions may lead to dental mobility, the risk of it might be as well reduced. Our study showed a significant result correlating the use of interdentally cleaning brushes and mobility: 50% of the women who often use interdentally cleaning brush had no dental mobility; the remaining 50% also had low teeth mobility.

In the end, it is important to remark the importance of regular dental checkups, which is highlighted by the high percentages of the women reporting teeth mobility and never going to the dentist (66.7%), opposite to the higher percentages of the women not reporting it and regularly undergoing dental checkups; this statistically significant *p* -Value ( *p* = 0.006) suggests how the correlation between the low presence of dental mobility and regular checkups is not casual, but supports the fact that regular visits and professional oral health care can positively affect and benefit oral health status.

Finally, although the values are not statistically significant, there is also increasing bleeding and bad breath in the ones who brush their teeth only once, when compared with the ones who brush their teeth at least three times a day, strengthening once again how important are proper and regular oral hygiene habits.

## Conclusion

In conclusion, this study highlights how more awareness needs to be raised concerning oral hygiene habits and diet, and also regarding the often-underestimated importance of regular dental checkups. Furthermore, not only brushing at least twice or three times a day needs to be encouraged but also the valid support of dental aids has to be in the everyday domestic oral hygiene protocol, as scientific evidence demonstrates.

A thorough anamnesis regarding the diet should be performed during dental visits, followed by proper nutritional advice related to oral health influence. Future clinical studies need to be performed on a more consistent number of vegetarian and vegan patients, to obtain more statistically significant results and support future research that will compare omnivorous, vegetarian, and vegan diets and their influence on oral health status. Furthermore, more variables that could influence results should be included in future studies, such as socioeconomic status, education, and other variables that could have impacted access to dental care and have influenced dental habits.
